# The +37 kb *Cebpa* Enhancer Is Critical for *Cebpa* Myeloid Gene Expression and Contains Functional Sites that Bind SCL, GATA2, C/EBPα, PU.1, and Additional Ets Factors

**DOI:** 10.1371/journal.pone.0126385

**Published:** 2015-05-04

**Authors:** Stacy Cooper, Hong Guo, Alan D. Friedman

**Affiliations:** Division of Pediatric Oncology, Johns Hopkins University School of Medicine, Baltimore, Maryland, United States of America; University of Cologne, GERMANY

## Abstract

The murine *Cebpa* gene contains an evolutionarily conserved 453 bp enhancer located at +37 kb that, together with its promoter, directs expression to myeloid progenitors and to long-term hematopoietic stem cells in transgenic mice. In human acute myeloid leukemia cases, the enhancer lacks point mutations but binds the RUNX1-ETO oncoprotein. The enhancer contains the H3K4me1 and H3K27Ac histone modifications, denoting an active enhancer, at progressively increasing levels as long-term hematopoietic stem cells transition to granulocyte-monocyte progenitors. We previously identified four enhancer sites that bind RUNX1 and demonstrated that their integrity is required for maximal enhancer activity in 32Dcl3 myeloid cells. The +37 kb *Cebpa* enhancer also contains C/EBP, Ets factor, Myb, GATA, and E-box consensus sites conserved in the human +42 kb *CEBPA* enhancer. Mutation of the two C/EBP, seven Ets, one Myb, two GATA, or two E-box sites reduces activity of an enhancer-promoter reporter in 32Dcl3 cells. In 293T gel shift assays, exogenous C/EBPα binds both C/EBP sites, c-Myb binds the Myb site, PU.1 binds the second Ets site, PU.1, Fli-1, ERG, and Ets1 bind the sixth Ets site, GATA2 binds both GATA sites, and SCL binds the second E-box. Endogenous hematopoietic RUNX1, PU.1, Fli-1, ERG, C/EBPα, GATA2, and SCL were previously shown to bind the enhancer, and we find that endogenous PU.1 binds the second Ets site in 32Dcl3 cells. Using CRISPR/Cas9, we developed 32Dcl3 lines in which the wild-type enhancer alleles are replaced with a variant mutant in the seven Ets sites. These lines have 20-fold reduced *Cebpa* mRNA when cultured in IL-3 or G-CSF, demonstrating a critical requirement for enhancer integrity for optimal *Cebpa* expression. In addition, these results indicate that the +37 kb *Cebpa* enhancer is the focus of multiple regulatory transcriptional pathways that impact its expression during normal hematopoiesis and potentially during myeloid transformation.

## Introduction

CCAAT/enhancer binding protein α (C/EBPα) is a basic region-leucine zipper transcription factor expressed preferentially within granulocytic and monocytic myeloid cells during hematopoiesis [1]. C/EBPα levels increase as long-term hematopoietic stem cells (LT-HSC) differentiate to the common myeloid progenitor (CMP) and subsequently to the granulocyte-monocyte progenitor (GMP), with *Cebpa* gene deletion reducing LT-HSC quiescence and preventing GMP formation [2–5]. Beyond the GMP stage, high-level C/EBPα is required for granulopoiesis and reduced levels are necessary for monopoiesis [6].

C/EBPα expression or activity is commonly diminished in acute myeloid leukemia (AML) cases, with *CEBPA* open reading frame point mutations impacting trans-activation or DNA-binding, RUNX1-ETO reducing *CEBPA* transcription, and other alterations such as those leading to Trib2 over-expression potentially also impeding C/EBPα expression, and thereby myeloid differentiation, to contribute to myeloid transformation [7–9].

The *Cebpa* promoter is auto-activated by C/EBPα and contains two near-consensus RUNX1 *cis* elements that bind RUNX1 in gel shift assay and chromatin immunoprecipitation (ChIP) assays and are functional in the 32Dcl3 myeloid cell line [10,11]. In addition, we identified a 453 bp segment centered at +37.5 kb in the murine *Cebpa* gene, having 85% identity with a homologous element at +41.8 kb in the human *CEBPA* locus, harboring enhancer specific H3K4me1 histone marks and capable of directing high-level hCD4 transgene expression to GMP [11,12]. The *Cebpa* enhancer binds endogenous RUNX1 as assessed by ChIP and contains four perfect RUNX1 consensus sites that bind RUNX1 in gel shift assay; mutation of these sites obviated 6-fold enhancer induction of promoter activity in 32Dcl3 cells [11]. RUNX1, as well as C/EBPα, PU.1, ERG, Fli-1, GATA2, SCL, Meis1, and Gfi-1b, had been found to bind chromatin in the region of this enhancer in hematopoietic cells, as determined by ChIP-Seq [13,14].

We have now sought to delineate additional +37 kb *Cebpa* enhancer functional elements, besides those that interact with RUNX1. We demonstrate that mutation of conserved C/EBP, Ets, Myb, GATA, and E-box sites each reduce enhancer activity in 32Dcl3 cells and utilize gel shift assays to show that C/EBPα, PU.1, Fli-1, ERG, Ets1, c-Myb, GATA2, and SCL have the capacity to bind one or more of their consensus sites within the enhancer and that PU.1 is the most prominent Ets factor that binds the enhancer in 32Dcl3 cells. Mutation of its seven Ets sites had the greatest consequence for enhancer activity in transient assays. We therefore also used CRISPR/Cas9 technology to replace endogenous enhancer alleles with a variant carrying these Ets site mutations, finding, on average, 20-fold reduced C/EBPα expression compared to parental cells. Further indicating the importance of the +37 kb enhancer for myeloid *Cebpa* gene regulation, analysis of previously reported global hematopoietic ChIP-Seq data, focusing on the *Cebpa* locus, demonstrates steadily increasing H3K4me1 and H3K27Ac histone modifications, indicative of an active enhancer, surrounding the +37 kb enhancer as LT-HSC progress GMP.

## Methods

### Transient Transfection

497 bp DNAs, containing the 453 bp enhancer and 21 upstream and 23 downstream bps with clustered point mutations, were synthesized (Blue Heron) and ligated as *Kpn*I/*Not*I fragments upstream of the -720/+125 *Cebpa* promoter in similarly digested *Cebpa*-Prom-Luc [11]. In 5’ to 3’ order within the enhancer, the two C/EBP sites TTCTGAAAT and CTTGCCACA were changed to ATGTCATAT and CTAGGCACA; the seven Ets sites GGAAG, CTTCC, GGAAG, CTTCC, CTTCC, AGGAAA and TTCC were changed to GGTTG, CAACC, CCAAG, CTTGG, CTTGG, AGGTTA and TTGG; the Myb site CAGTTA was changed to GGGTTA; the two GATA sites TTATCA and AGATA were changed to TTATGC and GTATA; and the two E-boxes CAAGTG and CACGTG were changed to GTAGTG and GTCGTG, with mutant bases underlined. The wild-type (WT) Enh/Prom-Luc reporter and a variant with point mutations in the four RUNX1 sites were previously described [11]. 32Dcl3 murine myeloid cells [15] were cultured in Iscove’s Modified Dulbecco Medium (IMDM) with 10% heat-inactivated fetal bovine serum (HI-FBS), 1 ng/mL murine IL-3 (Peprotech). 2E6 32Dcl3 cells were transiently transfected with 5 μg of these DNAs and 0.5 μg of CMV-βGal with DEAE—dextran, split 1:3 into IMDM/HI-FBS with IL-3 or 20 ng/mL human G-CSF (Amgen), and subjected to luciferase and β-galactosidase assays two days later as described [14].

### Gel Shift and ChIP Assays

293T cells (ATCC, CRL-3216) cultured in Dulbecco Modified Eagle’s Medium (DMEM) with 10% HI-FBS were transiently transfected with 6 μg CMV-C/EBPα, pECE-PU.1, MIG-Fli-1, MIG-ERG, CMV-Ets1, CMV-c-Myb, CMV-GATA2, or 3 μg MIG-SCL plus 3 μg pBabe-E12 in 100 mm dishes using 17 μL Lipofectamine 2000 (Invitrogen). Nuclear extracts were prepared two days later and gel shift and super-shift assays using these or 32Dcl3 nuclear extracts were performed, as described [16,17]. Oligonucleotide probes containing 5′-GATC overhangs were radio-labeled to similar specific activity with the use of Klenow enzyme and α-P^32^-dCTP. Sense strands of the WT probes used, with binding sites underlined, were as follows:

C/EBPa: 5’- GATCTACTTCCCGTTTCTGAAATCTGCCCCCA,

C/EBPb: 5’- GATCAGGTGACCTTCTTGCCACAACCACACATC,

ETSa: 5’- GATCCGGCGACCACAGGAAGTGCTGCCCTA,

ETSb: 5’- GATCTAGCTCAGTACTTCCCGTTTCTGAA,

ETSc: 5’- GATCTGGTGGCCAGGGAAGGCAGACTTGG,

ETSd: 5’- GATCCCAAGGCAGACTTCCCGCTGCCTCC,

ETSe: 5’- GATCACCCTGGGCTCTTCCCACCGGTCA,

ETSf: 5’- GATCTTATCAGAACAGGAAAGATGGCACCA,

ETSg: 5’- GATCCACACTCCTGTTCCCCACATCACA,

Myb: 5’- GATCAACCACACATCAGTTATTTATCAGAACA,

GATAa: 5’- GATCCATCAGTTATTTATCAGAACAGGAAA,

GATAb: 5’- GATCATGGCACCAGAGATATGTCCTCACC,

EBOXa: 5’- GATCCCACCGGTCACAAGTGGTTTGTTCCTG, and

EBOXb: 5’- GATCGTGTGGCTGGCACGTGCCAGCGGGGC.

Mutant versions of these probes contained point mutations corresponding to those introduced into the enhancer for transient transfection assays, as listed above. ChIP analysis of PU.1 in 32Dcl3 cells was conducted as described [11], utilizing 1E7 cells and 10 μg normal rabbit Ig or anti-PU.1 antiserum (T-21, Santa Cruz Biotechnology). Genomic DNA PCR primers were:

Cebpa-enh-F: 5’-AACAGGAAAGATGGCACCAG,

Cebpa-enh-R: 5’-CCACACCCCTCTATGTGATG,

Cebpa-40kb-F: 5’-CGCAATCAGGATGCACAATG, and

Cebpa-40kb-R: 5’-ACAGCCCGGCTGGCTTTC.

### CRISPR/Cas9 Homologous Targeting

Guide RNAs for Cas9-mediated genomic targeting minimizing off-target DNA cleavage were chosen using https://chopchop.rc.fas.harvard.edu/ [18], and corresponding DNA oligonucleotides were annealed and ligated into *Bbs*I digested pX330 (Addgene), which also expresses humanized *S*. *pyogenes* Cas9 (Addgene). The DNAs used to construct the CRISPR synthetic guide that led to successful targeting were:

CRISPR-F: 5’-CACCGCATGGCAAAATCAGAGGGG and

CRISPR-R: 5’-AAACCCCCTCTGATTTTGCCATGC.

A homologous replacement (HR) template plasmid was constructed by inserting a 940 bp 3’ genomic homology arm downstream and a 3020 bp 5’ homology arm upstream of the enhancer variant carrying point mutations in the seven Ets sites, in the pUC plasmid. The homology arms were obtained from a C57BL/6 genomic DNA BAC (CHORI) using recombineering technology [19]. The 5’ arm, ending with 5’-TTTCCCTCCTGG, lacked 13 bp, 5’-CTCAGGGATGCCA, located just upstream of the synthetic enhancer DNA, which begins with 5’-CCCCTCTGATTTTGCCATGC. A floxed PGK-Neo cassette was inserted between the enhancer and the 5’ homology arm. 5E6 32Dcl3 cells were subjected to Amaxa Nucleofection (program E-032, buffer nucleoV) with 0.5 μg pX330-CRISPR and 2 μg mETS-HR DNA. Two days later, cells were placed in 96 well dishes at 2E4 cells/mL with 1.2 mg/mL G418 (total). Selected subclones were transduced with pBabePuro-Cre as described [11], and pooled transductants selected with 2 μg/mL puromycin. Genomic DNA was isolated using the Blood and Cell Culture DNA midi kit (Qiagen) and subjected to PCR with

ETSF: 5’-TCAGTTATTTATCAGAACAGGTT,

3’genR: 5’-CCACCCAAATCTCCATCCTCCTG,

gapF: 5’-TCTTTCCCTCCTGGCTCAG,

EnhR: 5’-CCACACCCCTCTATGTGATG,

WTF: 5’-TCAGTTATTTATCAGAACAGGAA,

3’armR: 5’-CACTGAGTCCCCTGGAATAG,

5’armF: 5’-GTACCAGTCAAAGAATCATAAG, and

WTR: 5’-CATATCTCTGGTGCCATCTTT.

Amplified bands were subjected to agarose gel electrophoresis and visualization after ethidium bromide staining.

### Western Blot and Quantitative RNA Analysis

Total cellular proteins were subjected to Western blotting using CEBPα (14AA), PU.1 (T-21), Fli-1 (C-19) or Ets1(C-20) antiserum (Santa Cruz Biotechnology) and β-actin (AC-15, Sigma-Aldrich) antibody, and total cellular RNA was subjected to quantitative RT-PCR for *Cebpa*, *PU*.*1*, *Fli1*, *ERG*, or *Ets1* and *mS16*, the latter encoding a ribosomal protein, as described [11]. Primer pairs employed were:

Cebpa-F: 5’-TGGATAAGAACAGCAACGAG,

Cebpa-R: 5’-TCACTGGTCAACTCCAGCAC,

Pu.1-F: 5’-CAGAAGGGCAACCGCAAGAA,

Pu.1-R: 5’-GCCGCTGAACTGGTAGGTGA,

Fli-1-F: 5’-CGCCTACAACACAGAAGTGC,

Fli-1-R: 5’-CGTGAGGACTGGTCTGTATGG,

ERG-F:5’-CCACAAATGAGCGCAGAGTG,

ERG-R:5’-GCCATATTCTTTCACCGCCC,

Ets1-F: 5’-AGCCGACTCTCACCATCATC,

Ets1-R: 5’-CAAGGCTTGGGACATCATTT,

mS16-F: 5’-CTTGGAGGCTTCATCCACAT, and

mS16-R: 5’-ATATTCGGGTCCGTGTGAAG.

### Statistics

Means and standard errors are shown. The Student *t* test was used for statistical comparisons. Band intensities were quantified using National Institutes of Health ImageJ software. ChIP-Seq data was displayed using IGV 2.3 (Broad Institute).

## Results

### Multiple *Cebpa* +37 kb Enhancer Sites are Active in 32Dcl3 Myeloid Cells

**Fig 1:**
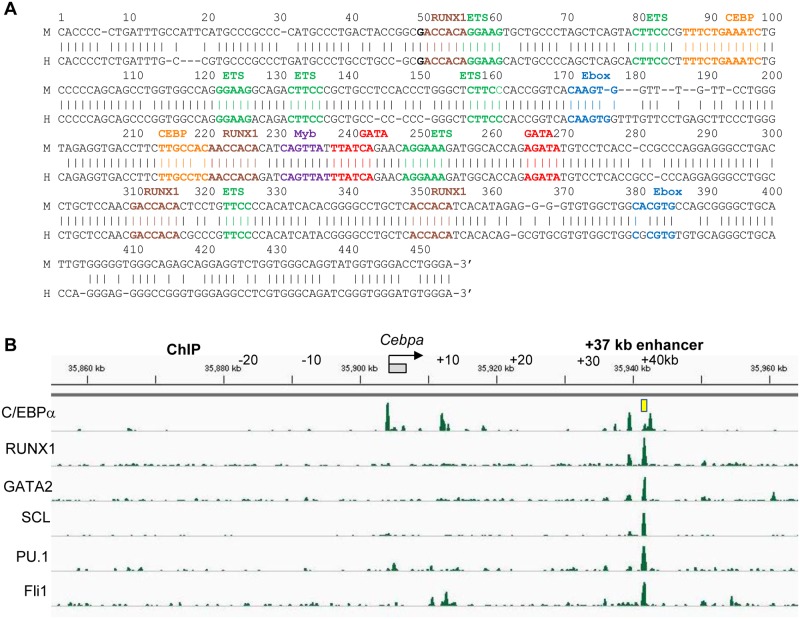
Predicted *cis* elements within the +37 kb *Cebpa* enhancer and binding of corresponding transcription factor within the *Cebpa* locus. **A**) Alignment of the +37 kb murine *Cebpa* and +42 kb human *CEBPA* enhancer sequences is shown, highlighting conserved GATA (consensus 5’-[A/T]GAT[A/C][A/G]), E-box (5’-CANNTG; SCL-binding), ETS (5’-[A]GGA[A/T][G/A]), C/EBP (5’-T[T/G]NNGNAA[T/G]), Myb (5’-[T/C]AAC[G/T]G), and RUNX1 [5’-PuACCPuCA] *cis* elements. **B)** ChIP-Seq data at the *Cebpa* locus in murine GMP and for RUNX1, GATA2, SCL, PU.1, and Fli-1 in the HCP7 murine stem cell line is shown [13, 14].

The murine +37 kb *Cebpa* and human +42 kb *CEBPA* enhancers are homologous over 453 bp and contain four consensus RUNX1, two consensus C/EBP, one consensus c-Myb, two consensus GATA, two consensus E-box, and seven consensus Ets sites (Fig 1A). ChIP-Seq data for C/EBPα in murine marrow GMP [14] demonstrates its binding at the *Cebpa* promoter and at +8 kb, +35 kb, and +37 kb, while ChIP-Seq data for RUNX1, GATA2, SCL, PU.1, and Fli-1 in the murine HPC7 myeloid stem cell line [13] demonstrates their binding predominantly at +37 kb and also at +35 kb (Fig 1B).

**Fig 2:**
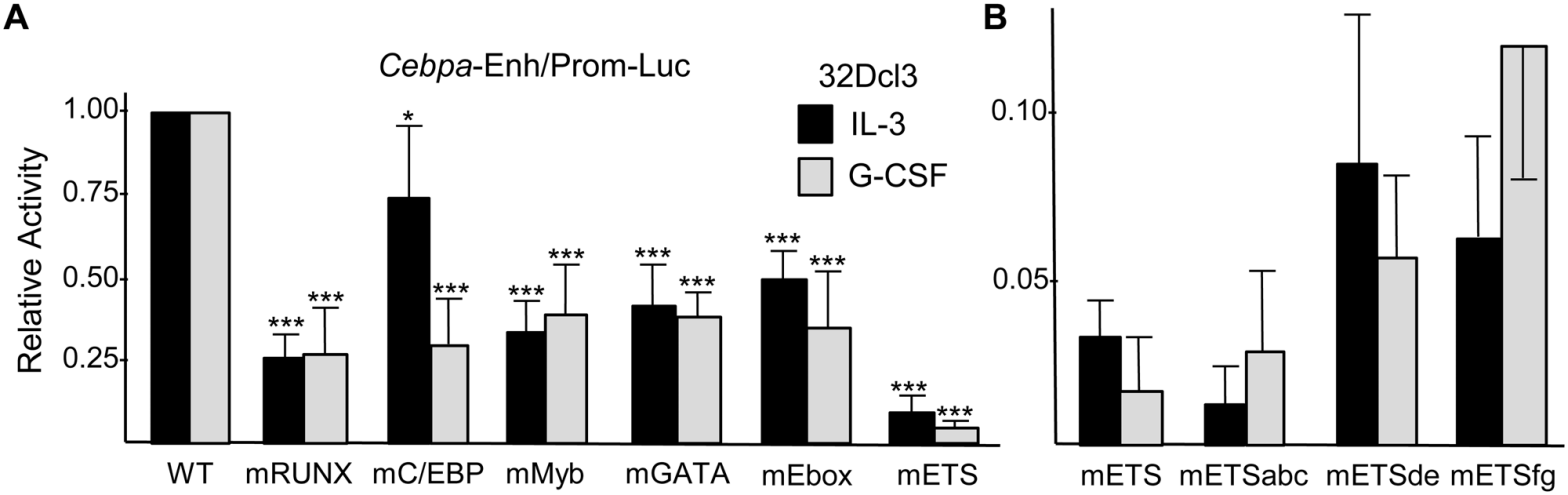
Mutation of conserved C/EBP, Myb, Ets, GATA, or E-box elements reduces +37 kb *Cebpa* enhancer activity in 32Dcl3 myeloid cells. **A**) *Cebpa* Enh/Prom-Luc constructs with either the wild-type (WT) *Cebpa* enhancer or variants mutant in four RUNX1 (mRUNX1), two C/EBP (mC/EBP), one Myb (mMyb), two GATA (mGATA), two E-box (mEbox), or seven Ets (mETS) sites were transiently transfected into 32Dcl3 myeloid cells with pCMV-βGal, which were then split into IL-3 or G-CSF media and assessed for luciferase and β-galactosidase activity two days later. Luciferase activity, normalized to β-galactosidase, for the WT construct was set to 1.0 in IL-3 or G-CSF in each experiment. The relative activities of the mutant constructs are shown (mean and SE from five determinations). **B**) The activities of mETS, mETSabc (mutant in the 5’ three Ets sites, mETSde (mutant in the central two Ets sites), and mETSfg (mutant in the 3’ two Ets sites) were assessed, relative to WT *Cebpa* Enh/Prom-Luc, in IL-3 and G-CSF (mean and SE from three determinations).

Variants of the murine +37 kb enhancer harboring clustered point mutations in the C/EBP, Myb, GATA, E-box, or Ets sites were synthesized, together with 23 upstream and 22 downstream base pairs, and positioned 5’ of the -720/+125 *Cebpa* promoter in pREP4-Luc to generate mC/EBP, mMyb, mGATA, mEbox, and mETS reporter plasmids. These DNAs, as well as related WT and mRUNX1 reporters studied previously [11], were transiently transfected into 32Dcl3 cells, together with CMV-βGal as internal control for transfection efficiency and extract yield. After transfection, cells were split between media containing IL-3 or G-CSF, the latter known to induce endogenous *Cebpa* transcription and granulocytic differentiation [1], and cell extracts were analyzed for luciferase and β-galactosidase activities 42 hr later (Fig 2A). Mutation of the four RUNX1 sites reduced activity 4-fold, on average, in both cytokines, similar to previous results [11]. Mutation of the two C/EBP sites reduced activity mildly in IL-3 and 4-fold in G-CSF, with greater effect in G-CSF likely reflecting increased endogenous C/EBPα. Introduction of 2 bp mutations into the single Myb site, the two GATA sites, or the two E-boxes reduced activity approximately 3-fold in IL-3 or G-CSF, and mutation of the seven Ets sites had the most dramatic effect, reducing activity >10-fold in each culture condition. A set of additional Enh/Prom-Luc reporters were then generated, mutating the 5’ three, central two, or 3’ two Ets sites to generate mETSabc, mETSde, and mETSfg reporters. Their activity was then evaluated in comparison with WT Enh/Prom-Luc and mETS-Prom-Luc in a separate set of experiments (Fig 2B). Each cluster of Ets site mutations greatly reduced reported activity, with mETSabc having the most marked, >20-fold reduction.

### C/EBPα, c-Myb, GATA2, SCL, and Ets Factors Bind Cognate *Cebpa* Enhancer Sites

**Fig 3:**
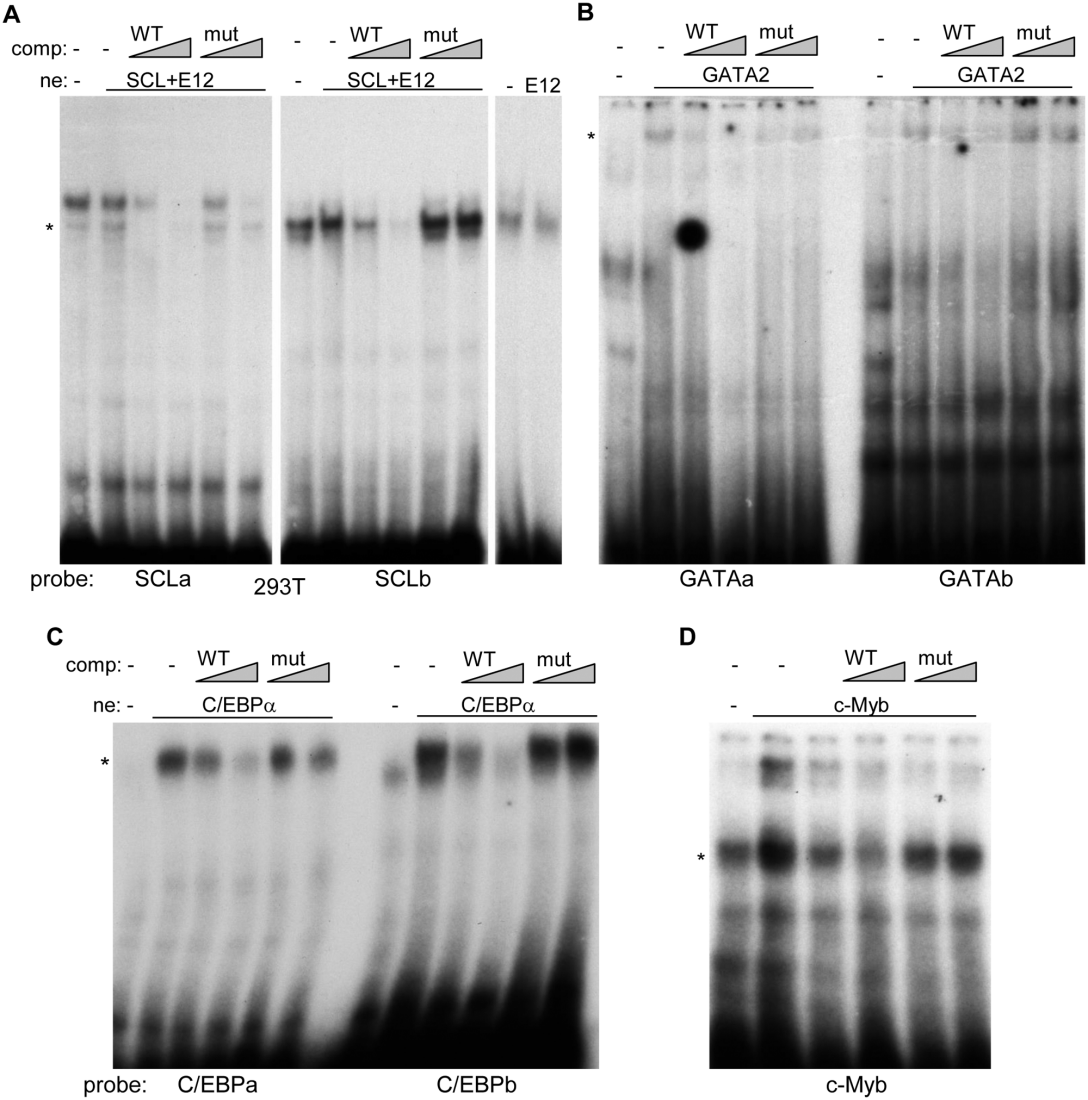
SCL, GATA2, C/EBPα, and c-Myb bind cognate *cis* elements within the +37 kb *Cebpa* enhancer. **A**) Nuclear extracts from 293T cells transiently transfected with vectors expressing SCL and E12, E12 alone, or empty vector, were subjected to gel shift analysis with radio-labeled SCLa or SCLb probes, corresponding to the upstream or downstream SCL *cis* elements, respectively. Binding of SCL+E12 was assessed in the presence of no competitor or 5- or 25-fold excess of WT or mutant non-radioactive competitor, the latter with point mutations in the central SCL motifs. *denotes specific bands. **B**) Nuclear extract expressing GATA2 was evaluated similarly for binding to the upstream GATAa or downstream GATAb consensus sites. **C**) Nuclear extracts from 293T cells transiently transfected with a vector expressing C/EBPα, or empty vector, were subjected to gel shift analysis with radio-labeled C/EBPa or C/EBPb probes, corresponding to the upstream or downstream C/EBP *cis* elements, respectively. Binding of C/EBPα was assessed in the presence no competitor or 5- or 25-fold excess of WT or mutant non-radioactive competitor, the latter with point mutations in the central C/EBP motifs. **D**) A nuclear extract expressing c-Myb was evaluated similarly for binding to the Myb consensus site.

We next sought to determine the affinities of transcription factors for their cognate consensus sites in the +37 kb *Cebpa* enhancer. Nuclear extracts were prepared from 293T cells transiently transfected with vectors expressing SCL and its heterodimeric partner E12, with GATA2, or with empty vector DNA. These were subjected to gel shift assay with radio-labeled, double-stranded 33–35 bp probes containing their predicted binding sites within the *Cebpa* enhancer. Gel shift reactions containing SCL/E12 or GATA2 included no competitor, 5- or 25-fold excess of unlabeled wild-type probe, or 5- or 25-fold excess of unlabeled probes mutant in the predicted protein binding sites (Fig 3A and 3B). As indicated in the figure legend, *denotes specific bands. The SCL+E12 extract increased binding to the SCLb site above background, whereas little or no increase was seen with the SCLa probe (compare lanes 1 and 2 of each gel), and WT but not the mutant non-radioactive probes competed effectively for binding in each case (lanes 3–6), demonstrating specific binding at the centrally located consensus E-box sites. As E12 could potentially dimerize with endogenous bHLH factors, we also evaluated binding of exogenous E12 alone, finding no increase above basal levels (Fig 3A, right panel). Weak but specific binding of GATA2 was seen also seen at the GATA consensus sites within both the GATAa and GATAb probes.

We also generated 293T cell nuclear extracts expressing C/EBPα or c-Myb, both known to bind and activate multiple myeloid-specific genes, include those encoding myeloperoxidase (MPO), neutrophil elastase (NE), or the G-CSF receptor [17,20,21]. In gel shift assay, C/EBPα specifically bound both the C/EBPa and C/EBPb probes, with greater affinity for the more downstream C/EBPb site (Fig 3C). Exogenous c-Myb increased binding above background levels to the radio-labeled Myb site probe, with binding inhibited effectively by excess WT but not the mutant Myb oligonucleotide (Fig 3D).

**Fig 4:**
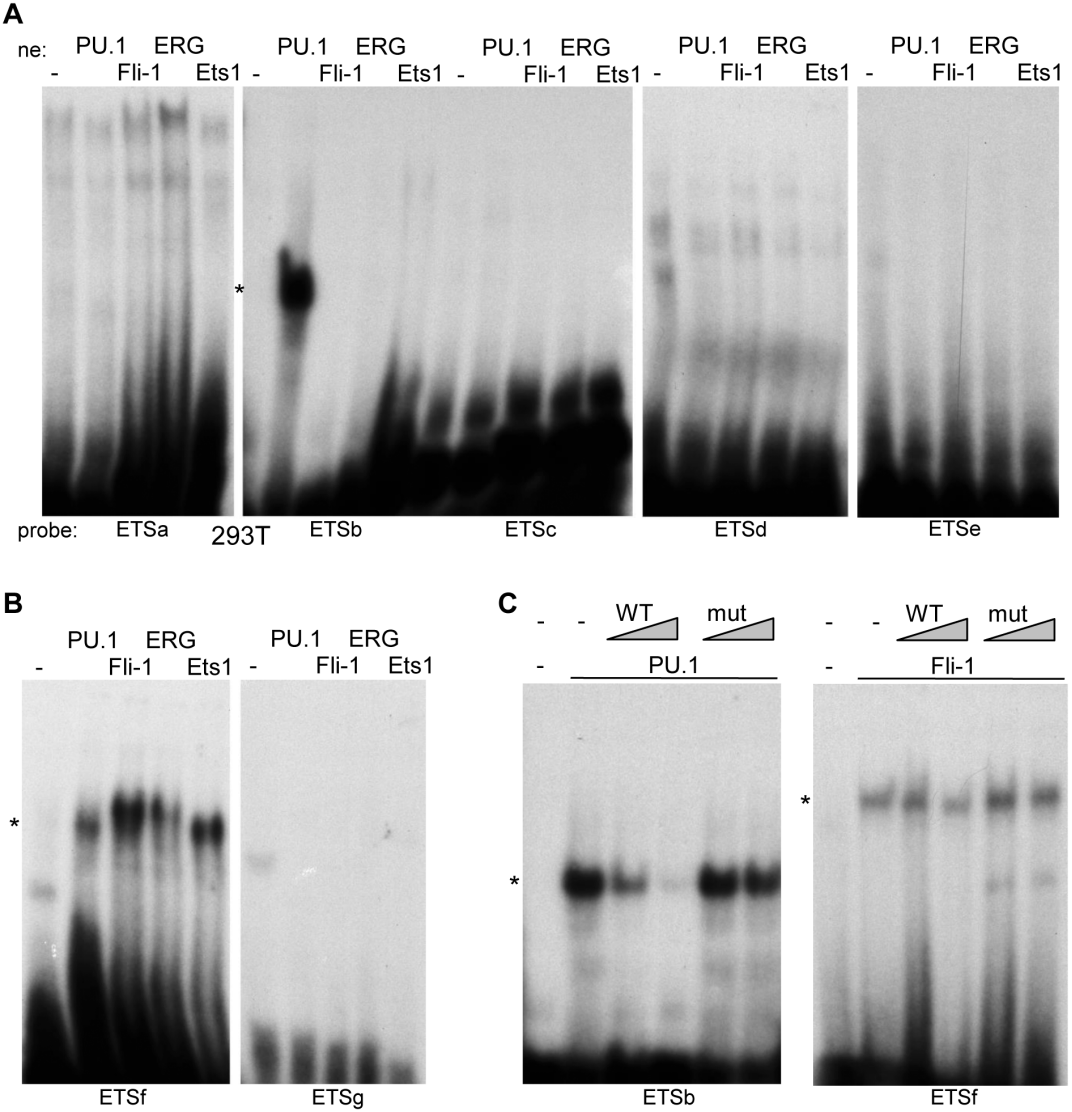
PU.1, Fli-1, ERG, and Ets1 bind cognate *cis* elements in the +37 kb *Cebpa* enhancer. **A**) 293T cell nuclear extracts expressing PU.1, Fli-1, ERG, or Ets1, or a control extract, were assessed for binding to radio-labeled ETSa, ETSb, ETSc, ETSd, or ETSe probes, containing the first to fifth Ets sites. **B**) Binding of these factors to the ETSf or ETSg probes, containing the sixth and seventh Ets sites, was assessed similarly. **C**) 293T nuclear extract expressing PU.1 was assessed for binding to the ETSb probe in the presence of no competitor or 5- or 25-fold excess WT or mutant competitor (left panel), and nuclear extract expressing Fli-1 was assessed similarly for binding to the ETSf probe (right panel).

The Ets factors PU.1, Fli-1, and ERG were previously shown to bind the *Cebpa* +37 kb enhancer in ChIP-Seq assays [13], and RUNX1, which also binds the enhancer, cooperates with Ets1 in gene activation [22]. We therefore generated 293T extracts expressing PU.1, Fli-1, ERG, or Ets1 and compared their ability to bind radio-labeled oligonucleotides corresponding to the seven Ets sites. Strong binding of PU.1 to the ETSb probe was detected, no specific binding was seen to the ETSa, ETSc, ETSd, ETSe, or ETSg probes, and PU.1, Fli-1, ERG, and Ets1 each bound the ETSf probe (Fig 4A and 4B). Interaction of PU.1 with ETSb or Fli-1 with ETSf was effectively competed by excess unlabeled WT ETSb or ETSf DNAs but not by variants carrying a 2 bp mutation in their Ets consensus sites, indicating specific binding at these sites (Fig 4C).

### PU.1 Binds the *Cebpa* +37 kb Enhancer in 32Dcl3 Cells

**Fig 5:**
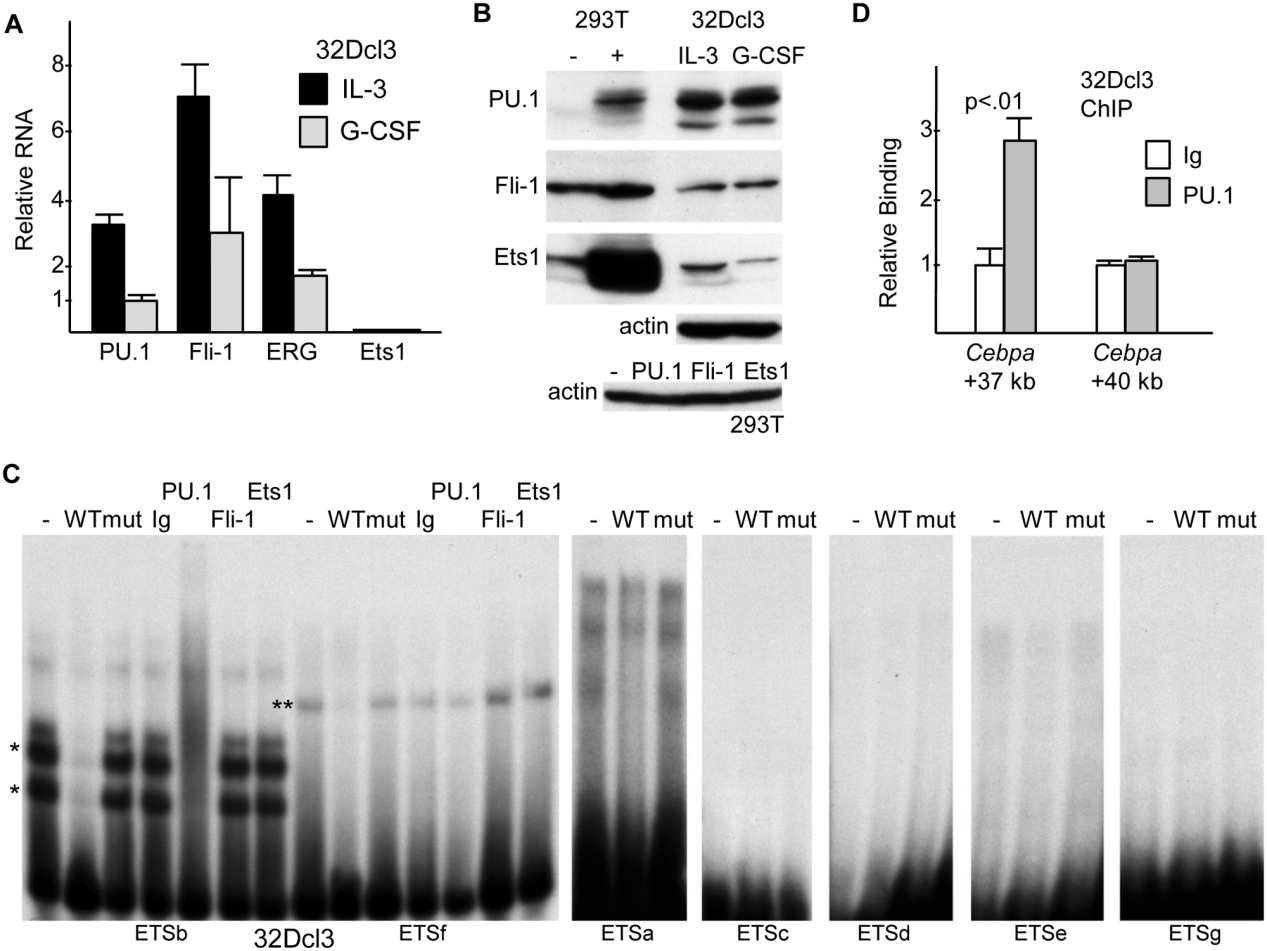
Endogenous PU.1 in 32Dcl3 myeloid cells binds the +37 kb *Cebpa* enhancer ETSb site. **A)** Total cellular RNAs from 32Dcl3 cells growing in IL-3 or after transfer to G-CSF for 24 hr were subjected to quantitative RT-PCR analysis for *PU*.*1*, *Fli-1*, *ERG*, and *Ets1*, relative to *mS16* murine ribosomal protein S16 RNA (mean and SE from three determinations). **B)** Total cellular proteins from 32Dcl3 cells grown in IL-3 or G-CSF and from 293T cells transfected two days earlier with empty vector or indicated expression vectors were subjected to Western blot analysis for PU.1, Fli-1, or Ets1 and β-actin. **C)** Nuclear extract obtained from 32Dcl3 cells proliferating in IL-3 was subjected to gel shift assays, including competition with 25-fold excess wild-type (WT) versus mutant (mut) unlabeled probes and super-shift analysis with 2 μg normal rabbit immunoglobulin (Ig) or 2 μg rabbit-anti PU.1, Fli-1, or Ets1 antisera, utilizing 1 ng radio-labeled probes ETSa-g. * and ** indicate specific gel shift species. **D)** 32Dcl3 cells in IL-3 were subjected to ChIP analysis utilizing Ig or PU.1 antiserum followed by quantitative PCR amplification of a 110 bp segment within the +37 kb *Cebpa* enhancer or a 140 bp segment located at +40 kb (mean and SE from three determinations).

To gain insight into whether these Ets factors might regulate the *Cebpa* +37 kb enhancer in 32Dcl3 cells, we first assessed their relative RNA and protein expression levels (Fig 5A and 5B). While PU.1, Fli-1, and ERG mRNA levels were within 3-fold of each other in IL-3 or G-CSF, Ets1 mRNA levels were 11,000-fold less in IL-3 and 4,000-fold less in G-CSF. Similarly, Western blot analysis demonstrated PU.1 levels several-fold higher, Fli-1 levels several-fold lower, and Ets1 levels markedly lower in 32Dcl3 cells than that achieved after transient transfection of their corresponding expression vectors into 293T cells. ERG protein levels could not be assessed due to lack of an available antibody that recognizes murine ERG.

We then conducted gel shift and super-shift analyses using 32Dcl3 nuclear extract and radio-labeled ETSa-g enhancer probes (Fig 5C). Strong binding to the ETSb probe was evident, manifested as two bands that were competed by WT but not mutant probe, and these bands were both super-shifted almost completely by PU.1 antiserum (left panel, lanes 1–7). Presence of two bands likely reflects partial proteolytic degradation in these protease-rich cells. The ETSf probe also yielded a shifted band that was competed by WT but not mutant ETSf competitor. This band had slower mobility than the ETSb PU.1 bands, had far weaker intensity even though the ETSf and ETSb probes were labeled to similar specific activity, and was not super-shifted by PU.1, Fli-1, or Ets1 antisera (left panel, lanes 8–14). The other five probes, ETSa, ETSc, ETSd, ETSe, and ETSg did not generate specific bands, although ETSa yielded a broad band with mobility similar to that obtained with ETSf. It is interesting to note that the pattern of binding of Ets factors present in 32Dcl3 nuclear extract to these probes is reminiscent of results obtained after exogenous Ets factor expression in 293T cells, where ETSb showed specific, strong binding to PU.1, ETSf bound all four Ets factors evaluated, albeit weakly to PU.1 relative to ETSb, and the other five sites did not manifest specific binding.

These gel shift data demonstrate that PU.1 present in 32Dcl3 cells binds the enhancer ETSb site, that an additional Ets factor that either has lower abundance, lower affinity, or both binds the ETSf site, and that this latter factor is distinct from PU.1, Fli-1, and Ets1. Based on these findings, we focused further on PU.1 and utilized the ChIP assay to determine whether endogenous PU.1 binds the +37 kb *Cebpa* enhancer in 32Dcl3 cells (Fig 5D). PU.1 antiserum enriched enhancer DNA 3-fold in comparison to normal rabbit Ig, whereas no enrichment was evident using primers that amplify a control DNA segment located 3 kb further upstream.

### CRISPR/Cas9-Mediated Endogenous *Cebpa* +37 kb Enhancer Ets Site Mutation Markedly Reduces *Cebpa* Expression

**Fig 6:**
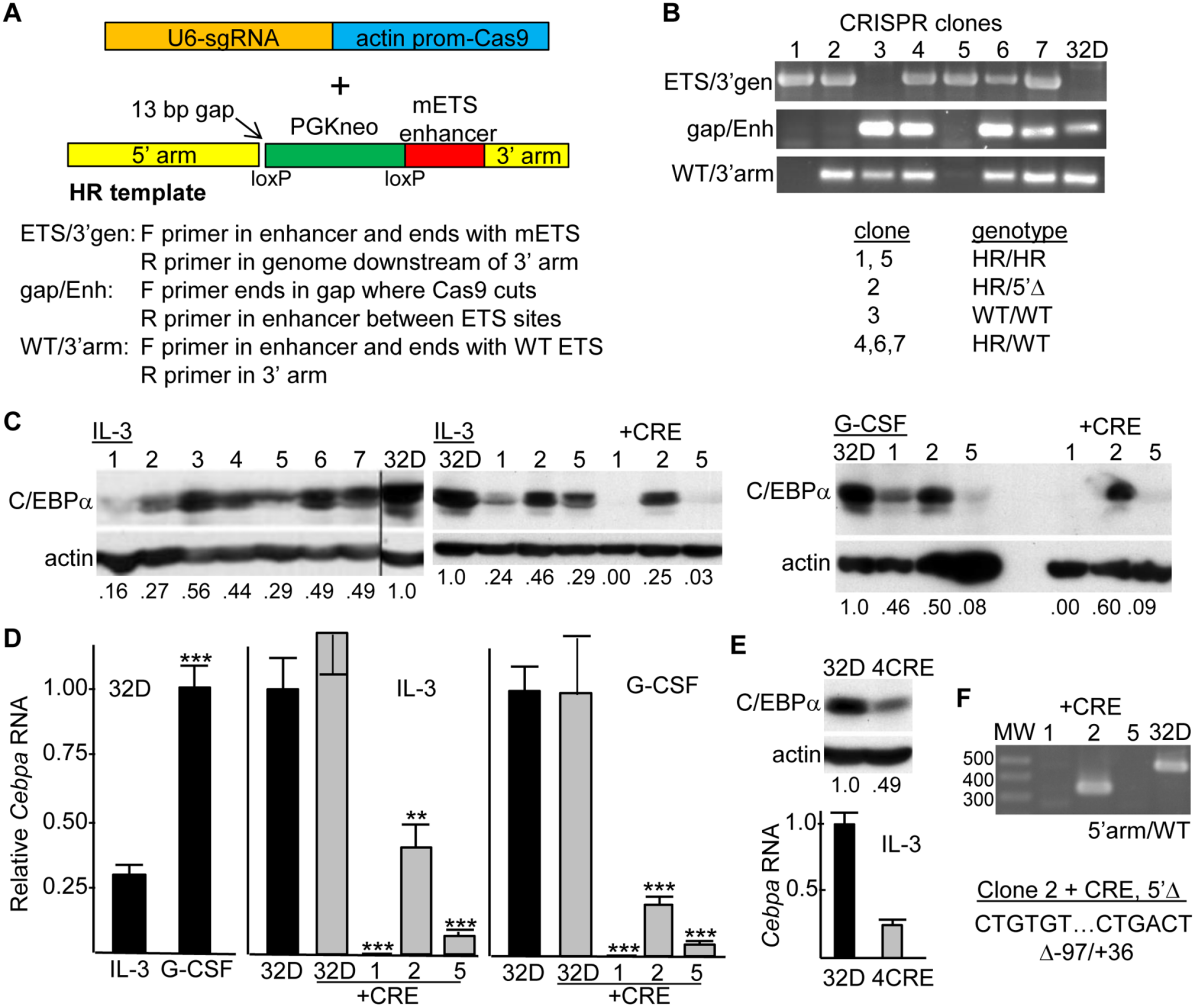
Homologous replacement of the WT with mETS *Cebpa* enhancer alleles reduces C/EBPα expression in 32Dcl3 myeloid cells. **A**) Diagram of a plasmid expressing a CRISPR synthetic guide RNA (sgRNA) from the U6 promoter and Cas9 from the actin promoter and a second plasmid containing the Homologous Replacement (HR) template. The mETS enhancer is linked directly to the 940 bp 3’ homology arm with an interposed *Not*I site but no sequence gaps, and a PGK-Neo cassette is interposed between the enhancer and a 3020 bp 5’ homology arm. A 13 bp sequence gap is present between the enhancer and the 5’ arm, to prevent the guide RNA, derived in part from this segment, from directing Cas9 to cut the HR template. Primers used for PCR of subclone DNAs are described. **B**) Genomic DNAs isolated from G418-resistant subclones isolated from 32Dcl3 cells nucleofected with the guide RNA/Cas9 and HR template plasmids were subjected to ETS/3’gen, gap/Enh, or WT/3’arm PCR. The expected 1.8 kb, 430 bp, or 392 bp PCR products were visualized by ethidium bromide staining after agarose gel electrophoresis (top). The genotype of each clone based on these results is summarized (bottom). HR—homologous replacement with mETS enhancer; WT—wild-type enhancer; 5’Δ—5’ enhancer deletion due to NHEJ. **C**) Total cellular proteins from these same cell lines, 2E5 cell equivalents per lane, were subjected to Western blotting for C/EBPα and β-actin (left). Parental 32Dcl3 cells, clones 1, 2, and 5, or clones 1/Cre, 2/Cre, and 5/Cre, cultured in IL-3 or for 24 hr in G-CSF, were evaluated similarly (middle, right). Numbers below each lane represent relative C/EBPα expression, normalized to β-actin, with expression in 32Dcl3 cells set to 1. **D**) Total cellular RNAs from parental 32Dcl3 cells, from 32D/Cre cells, or from clones 1/Cre, 2/Cre, and 5/Cre, cultured in IL-3 or for 24 hr in G-CSF, were analyzed for *Cebpa* expression, normalized to expression of the mRNA encoding ribosomal protein mS16 (mean and SE from three determinations). Shown is relative *Cebpa* expression in 32Dcl3 cells in IL-3 vs. G-CSF (left) or in all five lines in IL-3 (center) or G-CSF (right). **E)** Total cellular proteins from 32Dcl3 or 4/Cre cells cultured in IL-3 were subjected to Western blotting for C/EBPα and β-actin (top). Total cellular RNAs from these lines were analyzed for *Cebpa* expression (bottom, mean and SE from three determinations). **F**) Genomic DNA from clones 1/Cre, 2/Cre, and 5/Cre or 32Dcl3 cells was subjected to 5’arm/WT PCR, followed by gel electrophoresis and visualization by ethidium bromide staining together with molecular weight markers (MW). 32Dcl3 DNA yielded the expected 473 bp band. The shorter clone 2/Cre band was gel extracted and sequenced—the deleted bases, numbered relative to the 5’ end of the enhancer, are indicated.

Mutation of the seven Ets sites within the *Cebpa* enhancer almost completely eliminated Enh/Prom-Luc activity in 32Dcl3 myeloid cells. We next sought to determine the effect of introducing these same mutations into the endogenous enhancer. After initial screening for targeting efficacy, we selected a CRISPR synthetic guide RNA designed to specifically introduce double-stranded DNA breaks near the 5’ end of the *Cebpa* enhancer. We utilized plasmid pX330, which expresses the guide mRNA from a U6 promoter and Cas9 from the chicken β-actin hybrid (CBh) promoter. In addition, we constructed a homologous replacement (HR) template plasmid containing the mETS enhancer flanked by 3020 bp 5’ and 940 bp 3’ homology arms, with a floxed PGK-Neo cassette between the 5’ arm and the enhancer (Fig 6A). 13 bp normally present between the enhancer and 5’ arm were not included in the HR template, to prevent the guide mRNA from targeting the HR template or retargeting modified genomic alleles and to facilitate identification of successful targeting events. These two plasmids were transiently transfected into 32Dcl3 cells, followed by G418 selection in a 96 well format. Inclusion of the PGK-Neo cassette in the HR template and drug selection allowed us to enrich for subclones that underwent HR rather than non-homologous end joining repair (NHEJ) of introduced double-stranded breaks while also eliminating cells which experienced no genomic modification.

Seven G418-resistant 32Dcl3 subclones were subjected to three PCR reactions, as listed (Fig 6A). ETS/3’gen PCR utilized a forward primer ending with AGGTT-3’ from the mETSf site and a reverse primer 630 bp downstream of the enhancer, past the intervening microsatellite repeat. The forward primer will only amplify the mETS and not endogenous WT enhancer. A 1.8 kb band is expected if the HR template has replaced at least one of the two WT enhancer alleles. Such a band is seen in clones 1, 2, 4, 5, 6, and 7, but not clone 3 or parental 32Dcl3 cells (Fig 6B, top panel). Gap/Enh PCR utilized a forward primer with 3’ end CTCAG in the 13 bp gap between the 5’ arm and enhancer and a reverse primer located within the enhancer but not containing any of the seven mutant ETS sites. Absence of a PCR product would be consistent with biallelic replacement or disruption of the endogenous enhancer. DNA-derived from subclones 1, 2, and 5 lacked the predicted 430 bp PCR product, while subclones 3, 4, 6, and 7 and parental 32Dcl3 cells yielded a band of expected size on agarose gel electrophoresis (Fig 6B, middle panel). Finally, WT/3’arm PCR utilized a forward primer ending with CAGGAA from the wild-type ETSf site and reverse primer in the 3’ arm. Presence of a band indicates retention of at least one WT enhancer allele, and absence of a band indicates biallelic replacement with the mETS enhancer variant. Subclones 1 and 5 lacked the expected 392 bp band (Fig 6B, bottom panel). Together these PCR data indicate that subclones 1 and 5 have biallelic HR with the mETS enhancer, subclone 2 has on HR allele and one disrupted by NHEJ, expected to lead to a deletion in the 5’ enhancer region, subclone 3 has two WT enhancers, and subclones 4, 6, and 7 have one mETS and one WT allele, as summarized (Fig 6B).

The seven subclones and parental 32Dcl3 cells were subjected to Western blotting for C/EBPα and β-actin (Fig 6C, left panels). Clones 1, 2, and 5 had reduced C/EBPα relative to β-actin, as quantified with NIH ImageJ software. As PGK-Neo could itself affect *Cebpa* transcription, these 3 subclones were transduced with pBabePuro-Cre and pooled transductants isolated. Deletion of the floxed PGK-Neo cassette was evaluated by seeding these puromycin-resistant populations at 2E4 cell/mL in the absence or presence of G418. After 4 days, clone 1 or 5 cells expanded 70- or 135-fold in the absence of G418, whereas no viable cells were detected in its presence, indicating PGK-Neo deletion in >99% of the cells. Clone 2 expanded 54-fold in the absence of G418 and only 2-fold in its presence, indicating PGK-Neo deletion in >96% of the cells. Total cellular proteins from Cre-transduced clone 1, 2, and 5 cells were analyzed for C/EBPα and β-actin expression in comparison to non-transduced cells and parental 32Dcl3 cells, in IL-3 or 24 hr after transfer to G-CSF (Fig 6C, middle and right panels). Deletion of PGK-Neo led to even greater evident reduction in C/EBPα expression as a result of *Cebpa* +37 kb enhancer replacement with the mETS variant compared to parental cells, in particular for clones 1 and 5 that have biallelic replacement. Of note, clone 2, having one HR allele and one 5’-deleted allele, retains more C/EBPα protein than clones 1 and 5.

Total cellular RNAs from the three Cre-transduced subclones or from pooled Cre-transduced 32Dcl3 cells (grey bars), and from parental 32Dcl3 cells (black bars), were compared for *Cebpa* RNA expression, in IL-3 or G-CSF (Fig 6D). As seen previously [1], *Cebpa* RNA levels increased about 3-fold in response to G-CSF addition for 24 hr in parental cells (left graph), and in IL-3 or G-CSF clone 2/Cre had modest and clones 1/Cre and 5/Cre marked reduction in *Cebpa* RNA expression (center and right graphs). *Cebpa* RNA was expressed similarly in parental 32Dcl3 and 32D/Cre cells. For the two 32Dcl3 myeloid cell subclones with biallelic +37 kb *Cebpa* enhancer replacement with its mETS variant, *Cebpa* RNA was reduced on average 20-fold in either cytokine. For comparison, clone 4, having one HR and one WT allele, was transduced with pBabePuro-Cre, and expression of C/EBPα protein and mRNA was then compared to parental 32Dcl3 cells in IL-3 (Fig 6E). Mutation of one enhancer allele in this line led to 2-fold reduced C/EBPα protein and 4-fold reduced *Cebpa* mRNA, similar to results obtained with clone 2/Cre, which manifested 4-fold reduction in C/EBPα protein and 3-fold reduction in *Cebpa* mRNA.

To characterize the 5’ enhancer deletion induced by NHEJ repair in one of the clone 2 alleles, PCR of genomic DNA isolated from clones 1, 2, and 5 after Cre-mediated PGK-Neo deletion was conducted with a forward primer in the 5’ arm and reverse primer in the enhancer ending with TCTTT complementary to the wild-type ETSf site. Absence of the expected 473 bp band from clone 1/Cre and 5/Cre DNA confirms their having biallelic replacement with the mETS enhancer (Fig 6F). Clone 2/Cre DNA yielded a shorter band than that obtained from 32Dcl3 DNA, and sequencing of this band identified presence of a 133 bp deletion, including bps 1–36 of the enhancer and an additional 97 bp just upstream (Fig 6F). Although this 36 bp enhancer deletion does not impact any of the *cis* elements we have investigated, sequence conservation in this region suggests that additional transcription factors might bind here.

In summary, these data indicate that the +37 kb *Cebpa* enhancer plays a key role in stimulating *Cebpa* gene transcription in this myeloid cell line and that its ETS sites contribute substantially to endogenous enhancer activity.

### The *Cebpa* Enhancer Gains Activating Histone Marks as LT-HSC Develop into GMP

**Fig 7:**
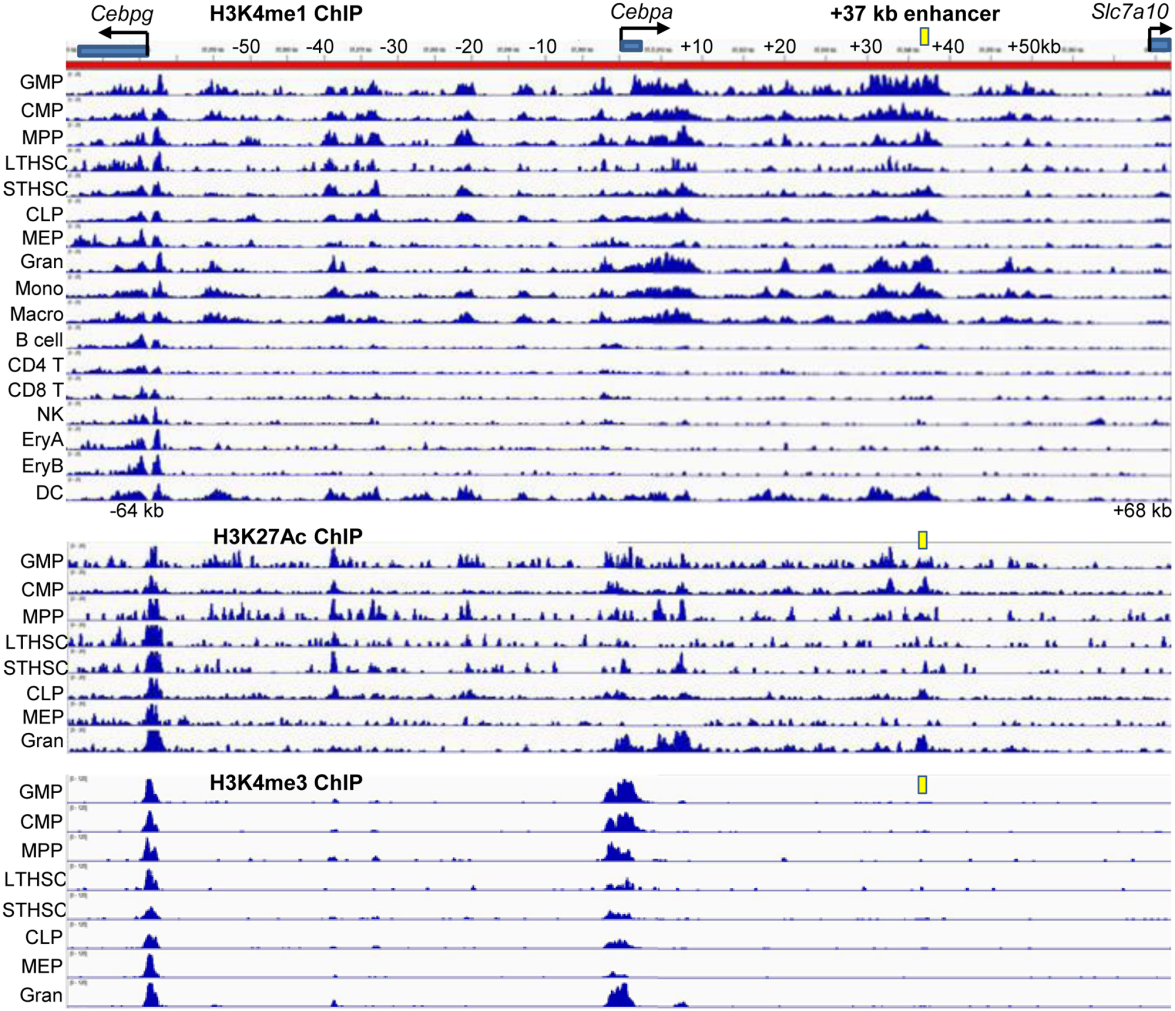
H3K4me1, H3K27Ac, and H3K4me3 histone modifications at the *Cebpa* locus in different murine hematopoietic subsets. ChIP-Seq data [23] was visualized with IVG 2.0 software (Broad Institute) for the granulocyte-monocyte progenitor (GMP), common myeloid progenitor (CMP), multi-potent progenitor (MPP), long-term hematopoietic stem cell (LT-HSC), short-term hematopoietic stem cell (ST-HSC), common lymphoid progenitor (CLP), megakaryocyte-erythroid progenitor (MEP), granulocyte (Gran), monocyte (Mono), macrophage (Macro), B cell, CD4 T cell, CD8 T cell, natural killer (NK) cell, erythroid precursors (EryA, EryB), and dendritic cell (DC) murine marrow subsets. The locations of the +37 kb enhancer (yellow box), the *Cebpg*, *Cebpa*, and *Slc7a10* transcription start sites (TSS, arrows), and the single exons that encode C/EBPγ or C/EBPα and the first exon of *Slc7a10* (grey boxes) are indicated.

To further support the importance of the +37 kb *Cebpa* enhancer in *Cebpa* gene regulation, we evaluated presence of the H3K4me1, H3K27Ac, and H3K4me3 histone modifications, indicative of poised enhancers, active enhancers, and active promoters, respectively, throughout the 132 kb murine *Cebpa* locus in various hematopoietic subsets (Fig 7), utilizing recently reported ChIP-Seq data [23]. Strikingly, both H3K4me1 and H3K27Ac increase at and surrounding the +37 kb enhancer as LT-HSC progress to the multipotent progenitors (MPP), the common myeloid progenitor (CMP), and finally the GMP; these activating marks are then maintained as GMP mature to granulocytes, monocytes, macrophages, and dendritic cells. The H3K4me3 at the *Cebpa* promoter follows the same expression pattern. The common lymphoid progenitor (CLP) retains intermediate levels of H3K4me1 and K3K27Ac at the +37 kb *Cebpa* enhancer and H3K4me3 at the *Cebpa* promoter, consistent with moderate expression of the *Cebpa* Enh/Prom-hCD4 transgene in the CLP [12]. However, these marks are lost in mature B cells, T cells, and NK cells and are minimal in the megakaryocyte-erythroid progenitor (MEP) and maturing erythroid cells, paralleling transgene and endogenous *Cebpa* expression [1,12]. Low, but detectable H3K4me1 and H3K27Ac at +37 kb and H3K4me3 at the *Cebpa* promoter in LT-HSC parallels transgene expression in FACS-defined LT-HSC [12].

In contrast, the promoter of the 3’ flanking gene, *Cebpg* contains constant levels of H3K4me3, H3K4me1, and H3K27Ac in each hematopoietic subset. The 5’ flanking gene, *Slc7a10*, encoding a neutral amino acid transport protein expressed only in neurons, is apparently inactive throughout hematopoiesis, as it lacks histone marks reflecting active transcription. Besides the increase of H3K4me1 and H3K27Ac seen in the 9 kb region surrounding the +37 kb *Cebpa* enhancer as LT-HSC progress to GMP, a similar pattern is seen between the *Cebpa* transcription start site (TSS) and +9 kb and to a lesser extent at -10 kb and -20 kb. In contrast, these marks are increased above baseline but remain constant at -33 and -40 kb, paralleling the pattern seen with the *Cebpg* promoter.

## Discussion

C/EBPα is a key regulator of normal myeloid development, and reduction of C/EBPα expression or activity, impeding differentiation, is a central feature of the majority of AML cases [9]. Therefore it is of interest to elucidate pathways regulating *Cebpa* gene expression during hematopoiesis. We previously demonstrated that a conserved 453 bp +37 kb genomic DNA element contains the enhancer-specific H3K4me1 mark and binds p300, that RUNX1 directly binds and increases enhancer activity, accounting for reduced C/EBPα and impaired granulopoiesis upon bialellic *Runx1* gene deletion, and that a *Cebpa* Enh/Prom-hCD4 transgene is expressed prominently in myeloid as compared to B lymphoid or erythroid progenitors, and also in FACS-defined or functional LT-HSC, but not outside of hematopoiesis [12]. Notably, although genomic sequencing of 110 human AML cases did not reveal point mutations or small insertions/deletions within the enhancer ([24] and T. Ley, personal communication), ChIP-Seq demonstrated that the RUNX1-ETO AML oncoprotein binds specifically at the homologous +42 kb *CEBPA* enhancer, but not the *CEBPA* promoter, in two patient samples and in the Kasumi-1 cell line [25].

In the present study, we extended previous ChIP-Seq data [13,14] to demonstrate that SCL, GATA2, C/EBPα, PU.1, Fli-1, ERG, and Ets1 bind the enhancer at specific consensus sites in gel shift assay, after exogenous expression in 293T cells, and that disruption of their cognate sites reduces enhancer activity in the 32Dcl3 myeloid cell line. In addition, we demonstrate that c-Myb also binds the enhancer in gel shift assay, and that mutation of its consensus site again reduces enhancer activity. ChIP-Seq data obtained using a murine marrow myeloid progenitor-derived cell line did not detect c-Myb binding at the +37 kb *Cebpa* enhancer, although binding at known sites in the MPO and NE promoters was evident [26]; therefore, a role for c-Myb in enhancer regulation must be considered less certain. Mutation of the Myb site could alternatively affect binding of other proteins within the enhancer. Mutation of three different clusters of putative Ets sites markedly reduced enhancer activity, though we only identified specific binding of PU.1 to the ETSb site and PU.1, Fli-1, ERG, and Ets1 to the ETSf site in 293T cells. Perhaps additional Ets factors or non-Ets factors bind near the ETSd or ETSe sites. Of note, the extended ETSb site, AAACGGGAAG, more closely matches the PU.1 consensus AAAG(A/C/G)GGAAG [27] than the other six enhancer Ets sites. And the ETSf site, GGAAA, is distinct from the ETSa-e, GGAAG, and ETSg, GGAAC, sites. Endogenous PU.1 present in 32Dcl3 cells bound the ETSb probe in gel shift assay and was found, using the ChIP assay, to interact with the +37 *Cebpa* enhancer. Among the other six enhancer Ets sites, only ETSf demonstrated specific interaction with endogenous proteins present in a 32Dcl3 nuclear extract, and super-shift and expression data suggest that these are distinct from Fli-1 or Ets1. Our findings reveal transcription factors beyond RUNX1 that activate the +37 kb *Cebpa* enhancer during normal hematopoiesis and which might be targeted directly or via regulatory pathways during myeloid transformation. Potentially, SCL, GATA2, RUNX1, Fli-1, and ERG enable low-level enhancer activity in LT-HSC, with induction of C/EBPα, PU.1, and c-Myb leading to increased enhancer activity in GMP.

Revealing a critical requirement for an intact +37 kb *Cebpa* enhancer for optimal *Cebpa* gene activity in myeloid cells, we find that biallelic homologous replacement with a variant harboring mutations in the seven Ets sites reduces C/EBPα expression 20-fold, on average, in two 32Dcl3 subclones cultured in IL-3 or G-CSF. In addition, analysis of published ChIP-Seq data demonstrated increased active H3K4me1 and H3K27Ac histone marks at and surrounding the enhancer as LT-HSC matured to MPP, CMP, and finally GMP. These marks were present as well in CLP, but only minimally MEP, and were absent in mature lymphoid and erythroid but retained in mature myeloid cells. Three other regions of the *Cebpa* locus, centered at -20 kb, -10 kb, and +1–9 kb also exhibited a similar pattern of increasing active histone modifications during progression of LT-HSC to GMP, suggesting that these regions may also harbor hematopoietic enhancers regulating *Cebpa* expression, though of note the +1–9 kb and +30–39 kb regions have the most prominent and broad increase in H3K4me1 and H3K27Ac, suggesting that these are more important than the -20 kb and -10 kb upstream regions in this regard. Related to this, we noted regions of homology between the murine and human *Cebpa* loci not only at +37 kb, but also at +2.5, +5, +8, and +35 kb, though only the +37 kb enhancer contains conserved RUNX1 sites [11]. As C/EBPα is expressed in multiple non-hematopoietic lineages, including hepatocytes, adipocytes, and type II pneumocytes, a subset of *Cebpa* genomic regions marked by H3K4me1 and H3K27Ac outside the +37 kb enhancer in GMP may also contribute to *Cebpa* gene activation in these cell types.

The *Cebpg* gene is located 64 kb upstream of *Cebpa*; its promoter region and DNA elements located at -33 and -40 kb relative to the *Cebpa* TSS, or +27 and +24 kb relative to the *Cebpg* TSS, show a constant level of activating histone modifications in LT-HSC, MPP, CMP, and GMP, suggesting that these elements regulate the *Cebpg* rather than the *Cebpa* gene. Perhaps the most important *Cebpa* regulatory elements are located downstream of the *Cebpa* TSS to avoid *Cebpg* gene activation, with an insulator element located between the two genes. Potentially related to this separation of regulatory elements, C/EBPα represses *Cebpg* gene activity [28]. The downstream *Slc7a10* gene is only expressed in neurons [29], and so is apparently not affected by the presence of the nearby +37 kb enhancer or other downstream *Cebpa* enhancers.

Active enhancers are transcribed bidirectionally, generating capped RNAs, and global analysis of such messages via cap analysis of gene expression or CAGE provides an additional means to identify active enhancers. When such analysis was conducted for the *CEBPA* locus in human neutrophils or monocytes, eight such putative enhancers were identified [30]. These are centered at -48 kb, -3 kb, +9 kb, +14 kb, +28 kb, +34 kb, +35 kb, and +43 kb. The 497 bp transcript generated by the latter enhancer is near the +42 kb human homolog of the murine +37 kb enhancer. The -48 kb regulatory element may control *CEBPG* rather than *CEBPA* gene expression. Notably, six of the remaining seven bidirectional transcripts are located downstream of the *CEBPA* TSS, consistent with the H3K4me1 and H3K27Ac ChIP data.

In summary, we provide further evidence supporting the importance of the +37 kb enhancer for *Cebpa* gene regulation during hematopoiesis, including binding by GATA2, SCL, PU.1, ERG, Fli-1, Ets1, C/EBPα, and potentially c-Myb, reduced activity in a myeloid cell line upon mutating their corresponding binding sites, markedly reduced *Cebpa* expression upon endogenous enhancer Ets site modification, and progressively increasing activating H3K4me1 and H3K27Ac histone marks as LT-HSC mature to GMP. These findings also implicate the homologous +42 kb *CEBPA* enhancer as important during human hematopoiesis and potentially as an important target of alterations contributing to myeloid transformation.
